# Hydroxyapatite, fluor-hydroxyapatite and fluorapatite produced via the sol–gel method: dissolution behaviour and biological properties after crystallisation

**DOI:** 10.1007/s10856-013-5050-y

**Published:** 2013-09-20

**Authors:** Christopher J. Tredwin, Anne M. Young, Ensanya A. Abou Neel, George Georgiou, Jonathan C. Knowles

**Affiliations:** 1 Plymouth University Peninsula Schools of Medicine and Dentistry, University of Plymouth, The John Bull Building, Tamar Science Park, Research Way, Plymouth, PL6 8BU UK; 2 Division of Biomaterials and Tissue Engineering, UCL Eastman Dental Institute, 256 Grays Inn Road, London, WC1X 8LD UK; 3 Division of Biomaterials, Conservative Dental Sciences Department, King Abdulaziz University, Jeddah, Saudi Arabia; 4 Biomaterials Department, Faculty of Dentistry, Tanta University, Tanta, Egypt; 5 WCU Research Centre of Nanobiomedical Science, Dankook University, San#29, Anseo-dong, Dongnam-gu, Cheonan-si, Chungnam 330-714 South Korea

## Abstract

Hydroxyapatite (HA), fluor-hydroxyapatite (FHA) with varying levels of fluoride ion substitution and fluorapatite (FA) were synthesised by the sol–gel method as possible implant coating or bone-grafting materials. Calcium nitrate and triethyl phosphite were used as precursors under an ethanol–water based solution. Different amounts of ammonium fluoride were incorporated for the preparation of the FHA and FA sol–gels. After heating and powdering the sol–gels, dissolution behaviour was assessed using ion chromatography to measure Ca^2+^ and PO_4_^3−^ ion release. Biological behaviour was assessed using cellular proliferation with human osteosarcoma cells and alamarBlue™ assay. Statistical analysis was performed with a two way analysis of variance and post hoc testing with a Bonferroni correction. Increasing fluoride substitution into an apatite structure decreased the dissolution rate. Increasing the firing temperature of the HA, FHA and FA sol–gels up to 1,000 °C decreased the dissolution rate. There was significantly higher cellular proliferation on highly substituted FHA and FA than on HA or Titanium. The properties of an implant coating or bone grafting material can be tailored to meet specific requirements by altering the amount of fluoride that is incorporated into the original apatite structure. The dissolution behaviour can further be altered by the temperature at which the sol–gel is fired.

## Introduction

Hydroxyapatite [HA, Ca_10_(PO_4_)_6_(OH)_2_] has attracted much attention for use in combination with Ti and its alloys, the aim being to combine bioactivity with mechanical strength [1–3]. Fluorapatite [FA, Ca_10_(PO_4_)_6_F_2_] has also gained much interest as pure form; FA is known to have a greater chemical stability and hence lower bioresorption rate than HA [4, 5]. There is potential that as HA forms fluor-hydroxyapatite [FHA, Ca_10_(PO_4_)_6_(OH_x_F_y_)] by selective substitution of OH^−^ with F^−^ it may be possible to alter the dissolution properties and biological properties of the material [5].

Previous studies have suggested that fluorapatite (Ca_10_(PO_4_)_6_F_2_) has a similar biocompatibility with HA in terms of its fixation to [1] bone and bone in-growth [6–10]. These studies used discs doped with various amounts of fluoride ion (F^−^) to investigate the effect of F^−^ content on osteoblastic cell behaviour. They concluded that FHA is biocompatible and that the amount of F^−^ affects cell attachment, proliferation, morphology and differentiation of osteoblastic cells, proposing this to be related directly to the release of the fluoride ions.

Use of the sol–gel technique is technically simple, cost effective and beneficial for coating complex shapes such as implants [11, 12] and our previous study [13] has shown that it is possible to fabricate HA, FHA and FA effectively using the sol–gel technique, furthermore this study showed that levels of fluoride ion substitution in the FHA structure can be precisely controlled. This study sought to investigate the potential biological properties of these novel FHA materials and compare their dissolution and biological behaviour.

## Materials and methods

### Preparation of HA sols

16.16 g of triethylphosphite (TEP [P(C_2_H_5_0)_3_], Aldrich, USA) was hydrolysed for 72 h in a mixture of 33.12 g of ethanol and 5.04 g of distilled water (P containing solution, VWR, UK). This mixture was then added to a solution of 39.36 g calcium nitrate [Ca(NO_3_)_2_.4H_2_0, Aldrich USA] in 15.12 g of distilled water. 25 ml of a 5 % w/v solution of ammonium hydroxide (NH_4_OH, VWR, UK) was added to the solution in order to improve gelation and subsequent formation of an apatite structure; the solution was allowed to react for 24 h and then aged for a further 24 h at room temperature.

### Preparation of FHA and FA sols

The FHA sols were prepared using various amounts of ammonium fluoride (NH_4_F, Aldrich, USA) in the P containing solution. The [P]/[F] molar ratios were 12, 6, 4 and 3 in order to have the corresponding compositions of Ca_10_(PO_4_)_6_F_0.5_OH_1.5_, Ca_10_(PO_4_)_6_F_1_OH_1_, Ca_10_(PO_4_)_6_F_1.5_OH_0.5_ and Ca_10_(PO_4_)_6_F_2_ (denoted by replacing the OH group with F ions in molar ratios of 0.25, 0.5, 0.75 and 1 respectively). After stirring for 72 h, the solutions were added slowly to a solution containing a stoichiometric amount (Ca/P ~ 1.67) of calcium nitrate [Ca(NO_3_)_2_.4H_2_0, Aldrich USA] following the protocol developed for the HA sols. 25 ml of a 5 % (wt/vol) solution of ammonium hydroxide (NH_4_OH, VWR, UK) was added to the solution in order to improve gelation and subsequent formation of an apatite structure.

### Dissolution properties

Samples of the Ca_10_(PO_4_)_6_(OH)_2_, Ca_10_(PO_4_)_6_F_0.5_OH_1.5_, Ca_10_(PO_4_)_6_F_1_OH_1_ Ca_10_(PO_4_)_6_F_1.5_OH_0.5_ and Ca_10_(PO_4_)_6_F_2_ sol–gels were heated in air to 600 °C for 2 h in a furnace (Lenton Furnace, Lenton Thermal Designs Limited, UK) with a ramp of 5 °C per minute, dwell time of 60 min and cool down rate of 10 °C per minute. To investigate the effect of heating Ca_10_(PO_4_)_6_(OH)_2_, Ca_10_(PO_4_)_6_F_1_OH_1_ and Ca_10_(PO_4_)_6_F_2_ sol–gels were heated to 600, 700, 800 and 1,000 °C using the same protocol. All powders obtained were subsequently ball milled and passed through a sieve to give a particle size of approximately 20 μm.

1.0 g of the ball milled samples was placed in a Specac pressing die set and pressed to 7 tons, and heated in air to 600 °C for 2 h in a furnace (Lenton). The 5 mm diameter solid samples were then placed in 100 ml of ultra-pure water in a sealed container, 10 ml of this was analysed using ion chromatography daily for the first 7 days and then at weekly intervals for a further 6 weeks for Ca ^2+^ [Dionex ICS–1000 (Dionex Ltd, Surrey, UK)] and PO_4_^3−^ [Dionex ICS–2500 Ion Chromatograpy system (Dionex Lts, Surrey, UK)] ion release. To ensure that there was no dilution effect there were 14 identical samples prepared for each run. All samples were analysed in triplicate and a mean and standard deviation was calculated. A Chromeleon^®^ software package was used for data analysis.

### Biological properties

Samples of the Ca_10_(PO_4_)_6_(OH)_2_, Ca_10_(PO_4_)_6_F_0.5_OH_1.5_, Ca_10_(PO_4_)_6_F_1_OH_1_ Ca_10_(PO_4_)_6_F_1.5_OH_0.5_ and Ca_10_(PO_4_)_6_F_2_ sol–gels were heated in air to 600 °C for 2 h in a furnace (Lenton Furnace, Lenton Thermal Designs Limited, UK) with a ramp of 5 °C per minute, dwell time of 60 min and cool down rate of 10 °C per minute. They were subsequently ball milled to a particle size of approximately 20 μm. To allow an appropriate flat surface for cell growth and analysis 0.5 g of the ball milled samples was subsequently placed in a Specac die (13 mm wide) and pressed to 4 tons, and heated in air to 600 °C for 2 h in a furnace (Lenton). The samples were made in triplicate for each powder type and polished to a 3 μm finish. Polished titanium discs were used as a control. All samples were sterilised at 200 °C in a hot air oven (Gallenkamp, Weiss, UK) for 3 h prior to commencement of the cellular work.

The sterilised samples were pre-treated by incubation in a growth medium [Dulbecco’s modified Eagles Medium (DMEM, Gibco), 10 % fetal calf serum, and 1 % penicillin and streptomycin solution (Gibco)] for 24 h at 37 °C humidified atmosphere incubator of 5 % CO_2_ in air.

Human Osteosarcoma (HOS) cells were obtained from the UCL Eastman Dental Institute archive. These cells were in cryogenic storage and had been previously obtained from a gingival tissue mucosal biopsy culture after obtaining ethical approval. These cells were used to obtain a preliminary estimate of biological compatibility of Ca_10_(PO_4_)_6_(OH)_2_, Ca_10_(PO_4_)_6_F_0.5_OH_1.5_, Ca_10_(PO_4_)_6_F_1_OH_1_ Ca_10_(PO_4_)_6_F_1.5_OH_0.5_ and Ca_10_(PO_4_)_6_F_2_ and to compare it with both Titanium discs and tissue culture plastic (TCP) as a positive control. The cells were seeded at a density of 5 × 10^3^ cells/disc and were cultured at 37 °C humidified atmosphere incubator of 5 % CO_2_ in air; in a growth medium. The medium was changed every 3 days.

The measurement of HOS proliferation was based on measuring the metabolic activity of these cells using alamarBlue™ assay. At 1, 3, and 7 days’ time point, the culture media was removed and the samples were washed three times with warm PBS. Five hundred microlitres of 10 % Alamar Blue™ in warm Hanks Balanced Salt Solution was then added to each well and incubated for 90 min. The fluorescence was then measured at 530 nm excitation, and 590 nm emission using a Fluroskan Ascent plate reader (Labsystems, Helsinki, Finland). The fluorescence measurement was repeated twice for each sample. A background fluorescence reading was also taken from 10 % Alamar Blue™ at each occasion.

In summary two readings were taken from each of the three samples in each group—giving a total of six readings per group. At the same time two fluorescence readings were taken from the wells containing only Alamar Blue™, these values were then subtracted from the sample readings to give a true value of fluorescence from the samples and thus cell proliferation that had occurred. The data was tabulated in Microsoft Excel Worksheet. Statistical package for social sciences (SPSS) Version 14 was used to analyze the data. A two way analysis of variance was undertaken. Initially the effect of the independent variables to fluorescence was determined both individually and in combination. This was followed by a post hoc multiple comparison tests using the Bonferroni correction. These were used to compare differences in fluorescence for the different materials at each time point i.e. if there was a difference in cellular proliferation on any of the materials at that time point.

Assessment of HOS cell attachment was conducted at the 7 day time point using scanning electron microscopy (SEM). Samples for SEM were overnight fixed with 3 % glutaraldehyde in 0.1 M sodium cacodylate buffer (Agar Scientific Ltd., Essex, UK) at 4 °C, then dehydrated in graded alcohol (20, 50, 70, 90, and 100 %). The dehydrated samples were critically dried in hexamethyldisilazane (HMDS, Taab Laboratories Ltd., Berkshire, UK) for 5 min, and then left to air dry. The dried samples were then mounted on aluminium stub; sputter coated with gold–palladium alloy, and viewed using JEOL 5410LV Scanning electron microscope (JEOL UK Ltd, UK).

## Results

### Ion release

**Fig. 1:**
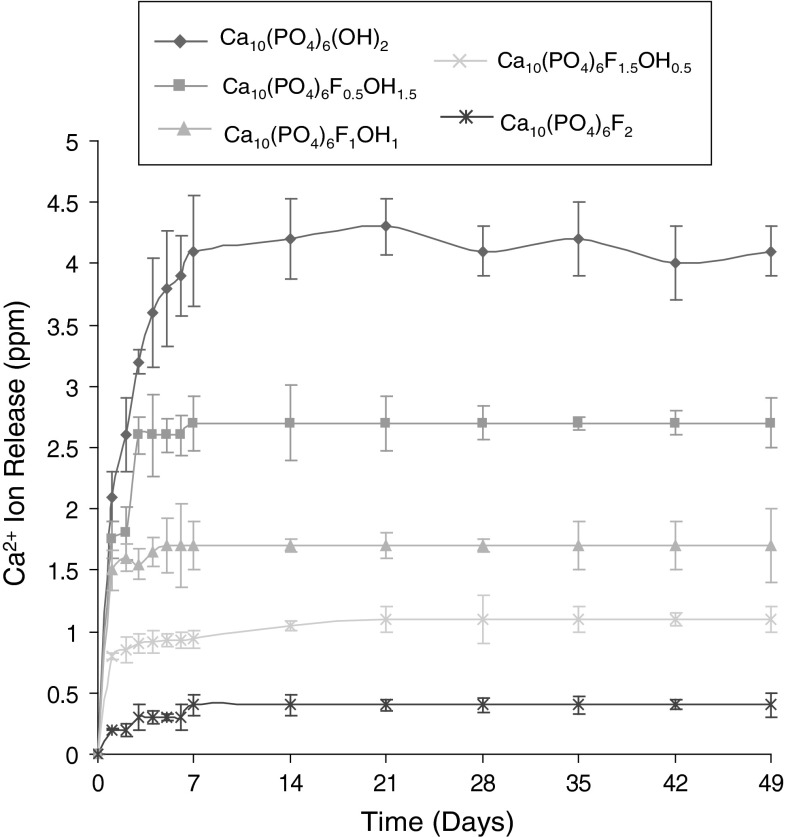
Calcium (Ca ^2+^) ion release from sol–gels heated to 600 °C, mean ± standard deviation

**Fig. 2:**
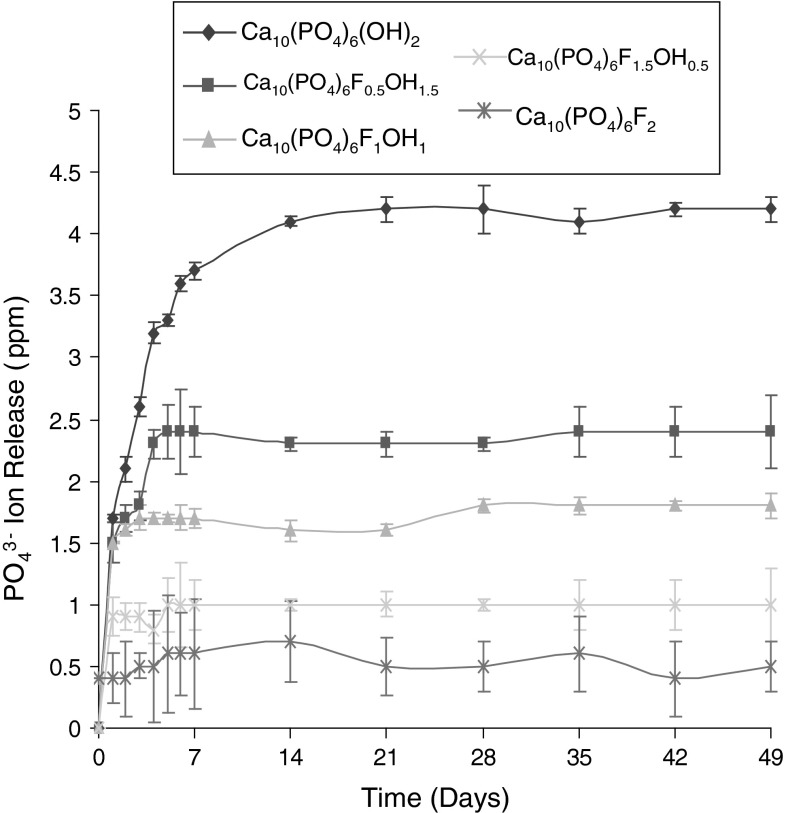
Phosphate (PO_4_^3−^) ion release from sol–gels heated to 600 ^o^C, mean ± standard deviation

The data obtained for the Ca ^2+^ and PO_4_^3−^ ion release is shown graphically in Figs. 1 and 2.

**Fig. 3:**
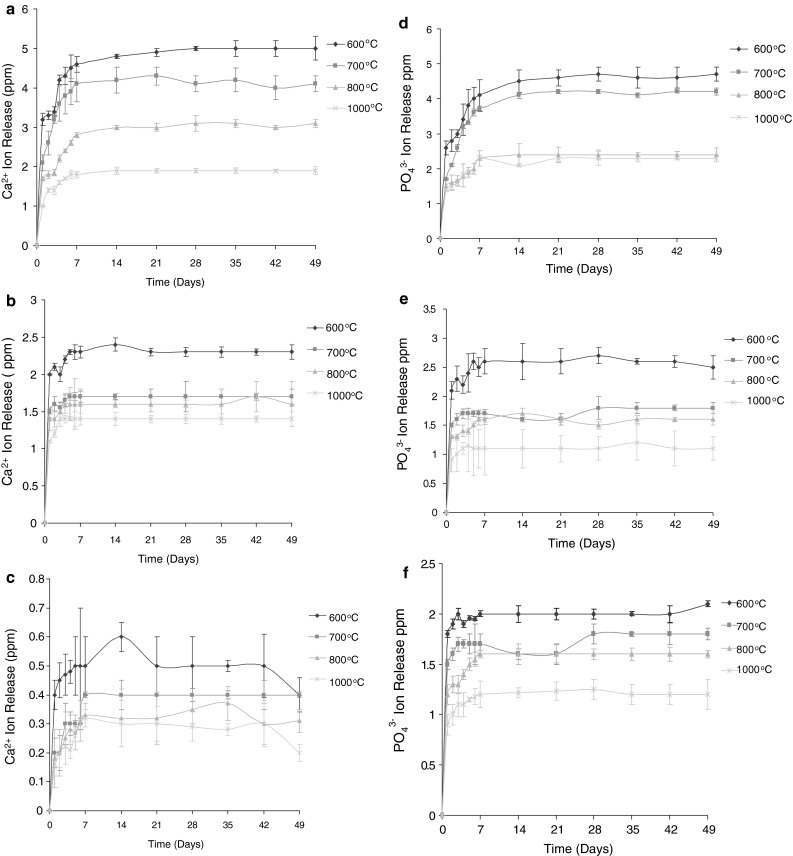
**a** *Graph* to show mean (±SD) Ca ^2+^ ion release for the Ca_10_(PO_4_)_6_(OH)_2_ produced sol–gel heated to 600, 700, 800 and 1,000 °C. **b** *Graph* to show mean (±SD) Ca ^2+^ ion release for the Ca_10_(PO_4_)_6_F_1_OH_1_ produced sol–gel heated to 600, 700, 800 and 1,000 °C. **c** *Graph* to show mean (±SD) Ca ^2+^ ion release for the Ca_10_(PO_4_)_6_F_2_ produced sol–gel heated to 600, 700, 800 and 1,000 ^°^C. **d** *Graph* to show mean (±SD) PO_4_^3−^ ion release for the Ca_10_(PO_4_)_6_(OH)_2_ produced sol–gel heated to at 600, 700, 800 and 1,000 ^°^C. **e** *Graph* to show mean (±SD) PO_4_^3−^ ion release for the Ca_10_(PO_4_)_6_F_1_OH_1_ produced sol–gel heated to at 600, 700, 800 and 1,000 ^°^C. **f** *Graph* to show mean (±SD) PO_4_^3−^ ion release for the Ca_10_(PO_4_)_6_F_2_ produced sol–gel heated to at 600, 700, 800 and 1,000 ^o^C

The data obtained for the Ca^2+^ ion release for the Ca_10_(PO_4_)_6_(OH)_2_, Ca_10_(PO_4_)_6_F_1_OH_1_ and Ca_10_(PO_4_)_6_F_2_ sol–gel heated to 600, 700, 800 and 1,000 °C is shown graphically in Fig. 3a, b, c, d, e and f. For the calcium release as a function of fluoride substitution, as can clearly be seen (Fig. 1), with increasing F^−^ substitution, there is a decrease in the amount of Ca^2+^ released. All curves show an initial faster release of calcium over the first 7 days and this then plateaus until the end of the experiment. This same trend is also seen for the PO_4_^3−^ release (Fig. 2). Figure 3a shows the effect of thermal treatment temperature of Ca_10_(PO_4_)_6_(OH)_2_ on calcium ion release. As expected, with increasing annealing temperature there is a decrease in the amount of calcium released. The samples also show the same initial faster release over the first 7 days. This same trend is seen in the PO_4_^3−^ release for this sample (Fig. 3d) and also for the other compositions (Fig. 3b,c, e and f).

### Biological behaviour

**Fig. 4:**
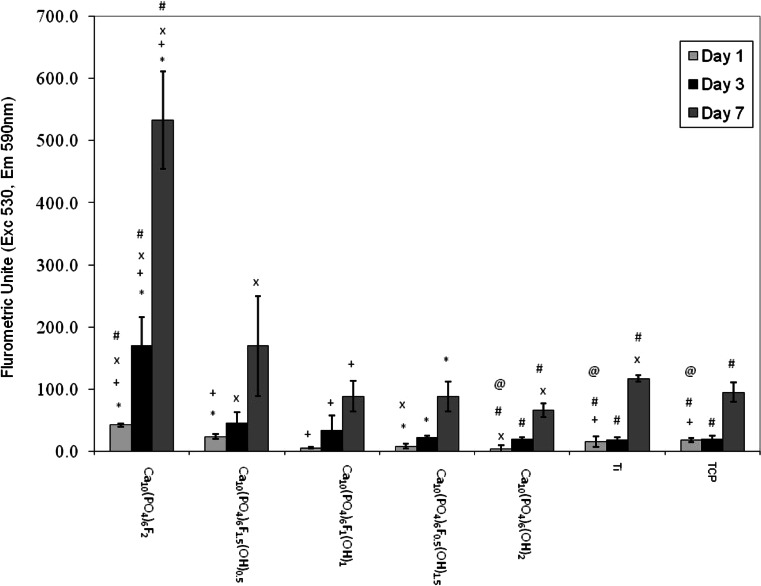
*Bar graph* to show mean (±SD) proliferation of Human Osteosarcoma Cells over a 7 day period. Statistical analysis key:^*^statistically significantly different from Ca_10_(PO_4_)_6_F_0.5_OH_1.5_ at that time point. ^+^statistically significantly different from Ca_10_(PO_4_)_6_F_1_OH_1_ at that time point. ^x^statistically significantly different from Ca_10_(PO_4_)_6_F_1.5_OH_0.5_ at that time point. ^#^statistically significantly different from Ca_10_(PO_4_)_6_F_2_ at that time point. ^@^statistically significantly different from Ca_10_(PO_4_)_6_OH_2_ at that time point

This data is represented graphically in Fig. 4 and there is clear trend for increased metabolic activity with increasing fluoride content, this was only actually significantly different with the highest concentrations of fluoride substitution but at this concentration it was statistically significantly different to all materials at all time points at *P* = 0.001. Ca_10_(PO_4_)_6_F_2_ showed significantly higher metabolic activity than HA at 1, 3 and 7 days, Ca_10_(PO_4_)_6_F_1.5_OH_0.5_ had significant higher cellular proliferation on days 1 and 7.

All cell numbers increased between day 1 and 7 indicating that the cells were able to increase their metabolic activity on all surfaces in the experimental protocol. The data showed trends in the metabolic activity with the different materials used. At each of the periods tested (1, 3 and 7 days) the metabolic activity values were significantly high for Ca_10_(PO_4_)_6_F_2_ (pure fluorapatite). Furthermore there was a general trend that can be seen very clearly in the graph in Fig. 4 that the higher the fluoride substitution that occurred in the apatite structure the higher the metabolic activity.

The statistically significant differences are denoted on Fig. 4. It is clear from this graph that Ca_10_(PO_4_)_6_F_2_ is statistically significantly different from all groups, including the control groups at all times points at *P* < 0.001.

## Discussion

### Dissolution behaviour

One of the most important issues when considering hydroxyapatite (HA) for biological coatings is the dissolution rate in an environment where human body fluids exist. It has been reported that plasma-sprayed HA coatings dissolve and degrade quickly, resulting in the weakening of the coating-substrate bonding or the implant fixation to the host tissues [11, 14, 15]. For this reason any changes to the HA that result in a higher chemical stability would be an advantage [16].

The ion release experimental methodology adopted, allows a possible insight to be gained of how the discs obtained may behave in vivo. Ca^2+^ and PO_4_^3−^ ion release were chosen to observe the dissolution behaviour of each disc since the apatite structure is known to collapse by the release of these ions.

For all the sol–gel prepared discs there was a clear pattern to the dissolution behaviour and release of Ca^2+^ and PO_4_^3−^ ions. The dissolution of all the discs showed an initial rapid increase over the first 7 days; this then stabilised to similar level for the next 7 weeks. Importantly, the fluoride substitution decreased the dissolution of the Ca^2+^ and PO_4_^3−^ ions, that is with the dissolution rate in the order Ca_10_(PO_4_)_6_(OH)_2_ > Ca_10_(PO_4_)_6_F_0.5_OH_1.5_ > Ca_10_(PO_4_)_6_F_1_OH_1_ > Ca_10_(PO_4_)_6_F_1.5_OH_0.5_ > Ca_10_(PO_4_)_6_F_2_.

Such a decrease in the dissolution rate of an apatite driven by the fluoride substitution can be potentially explained by the structural stability of each prepared disc. When considering the improvement in the crystallinity and crystallite size of the fluoride substituted discs [13, 16], such a retarded dissolution can be partially affected by the improvement in crystallisation. As a result of the observations it is deduced that the fluoride substitution effectively controls the dissolution rate of an apatite disc and suggest the possibility of tailoring the solubility of any coatings made from the sol–gels through fluoride ion substitution.

The dissolution behaviour and resultant Ca^2+^ and PO_4_^3−^ Ion release with different heating temperatures showed a similar profile for all the samples (albeit with different final values). There was a rapid increase in release of the PO_4_^3−^ ions over the first 7 days, this then stabilised to a similar level for the next 7 weeks. Importantly, the heating temperature was able to decrease the dissolution of the Ca^2+^ and PO_4_^3−^ ions with all the samples obtained, that is with the dissolution rate in the order 600 °C > 700 °C > 800 °C > 1000 °C.

Such a decrease in the dissolution rate of an apatite driven by the temperature to which the apatite has been heated can again be explained by the structural stability of each disc. Increasing the heating temperature from 600 to 1000 °C improves the crystallinity and decreases the crystal size of the HA/FHA/FA discs, suggesting that a retarded dissolution can be partially affected by the improvement in crystallisation. As a result of the observations it can be deduced that heating of the apatite structure from 600 to 1000 °C is effectively able to control the dissolution rate of an apatite disc and suggest the possibility of tailoring the solubility of any coatings made from the sol–gels through controlled heating.

### Cell proliferation

In this study it has been assumed that metabolic activity is indicative of changes in cellular growth numbers, with an increasing activity indicating cellular proliferation. The data shows that there was a clear trend for the fluor-hydroxyapatite and fluorapatite to outperform the hydroxyapatite coatings in terms of cellular proliferation.

Considering the known clinical biocompatibility of titanium and hydroxyapatite, the results of this study are very encouraging indeed. The hydroxyapatite [Ca_10_(PO_4_)_6_(OH)_2_] had significantly lower cellular proliferation on day 1 when compared with titanium, while on day 3 and 7 there was no significant difference. In contrast the cellular proliferation results for higher levels of substitution of the fluoride coating were significantly better than both titanium and hydroxyapatite. Postulating this forward to the clinical situation suggests that cellular proliferation and hence the first stages of healing could potentially be faster with fluoride substituted apatite coatings than with hydroxyapatite coatings or titanium itself.

Further encouragement from these results is that the higher level of fluoride substitution coatings performed significantly better than the tissue culture plastic. It must be understood that TCP is specifically designed to support and encourage cellular growth; for the Ca_10_(PO_4_)_6_F_2_ and Ca_10_(PO_4_)_6_F_1.5_OH_0.5_ to significantly outperform this shows great potential for use in vivo as a highly compatible material.

The results of this study provide support and build on the current knowledge in the literature. Fluorapatite coatings have attracted a great deal of attention in areas requiring long-term chemical and mechanical stability [17–19]. Previous studies have suggested that fluorapatite (Ca_10_(PO_4_)_6_F_2_) has a similar biocompatibility with HA in terms of its fixation to bone and bone in-growth [6–8]. Qu and Wei [10] studied discs doped with various amounts of fluoride ion (F^−^) to investigate the effect of F^−^ content on osteoblastic cell behaviour. They concluded that FHA is biocompatible and that the amount of F^−^ affects cell attachment, proliferation, morphology and differentiation of osteoblastic cells. The first study to suggest that fluorapatite may have superior biological properties to HA was Kim et al. [5]. This current study takes this work further and suggests that fluorapatite (Ca_10_(PO_4_)_6_F_2_) and highly substituted fluorhydroxyapatites (Ca_10_(PO_4_)_6_F_1.5_OH_0.5_) possibly have a superior biocompatibility to HA.

These fluoride substituted apatites produced by the sol–gel method in this study, have the potential to offer themselves as highly biocompatible titanium implant coating materials. Furthermore because of their high biocompatibility they could offer the potential as an alloplastic bone grafting material. Currently clinically one of the most commonly used bone grafting materials is BioOss™ (Geistlich, Austria) which is made from deproteinised bovine bone. It may be possible to produce a highly biocompatible osteoconductive material made from fluorapatite/fluoride substituted hydroxyapatite that could be used as a bone grafting material. The materials could be made in blocks or differing particle sizes and offer a range of properties depending on the level of fluoride substitution.

## Conclusions

1. Increasing levels of fluoride substitution into an apatite structure results in a structure that exhibits slower dissolution compared to that with lower or no fluoride substitution.
2. Increased firing temperature up to 1000 °C of hydroxyapatite and fluoride substituted hydroxyapatite results in a structure that exhibits slower dissolution compared to that with lower or no fluoride substitution.
3. Increasing the levels of fluoride substitution into an apatite structure results in increased biocompatibility.
